# Supplementary material for: Conserved neural circuit structure across Drosophila larval development revealed by comparative connectomics
Source: eLife. 2017 Oct 23;6:e29089. doi: 10.7554/eLife.29089 (PMC5662290; doi:10.7554/eLife.29089)
Supplement: Supplementary file 1. — For each cell type, we show a dorsal view (with CNS boundary, anterior up), a sagittal view (anterior to right), a cross-sectional view (grey line indicates neuropile boundary), and a table of number and fraction (in parentheses) of synapses from mdIV neurons onto the neuron shown. Due to varying anteroposterior extents of neurons, sagittal views are not to scale. [file elife-29089-supp1.pdf]

Local Neurons

A02m (1) - a1L

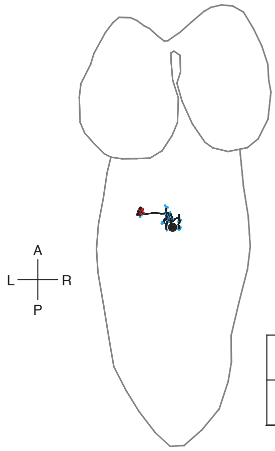

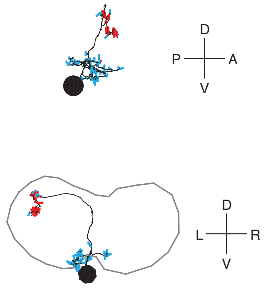

Inputs: #syn., (frac. syn)

	vdaB	v'ada	ddaC
Right	0 (0)	0 (0)	1 (0.0204)
Left	3 (0.0612)	2 (0.0408)	4 (0.0816)

A02m (1) - a1R

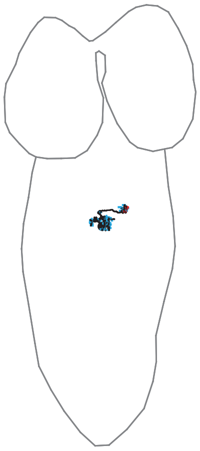

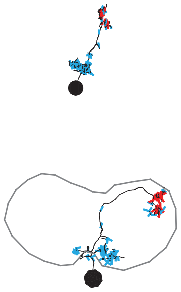

Inputs: #syn., (frac. syn)

	vdaB	v'ada	ddaC
Right	0 (0)	0 (0)	2 (0.026)
Left	0 (0)	0 (0)	1 (0.013)

A02m (2) - a1L

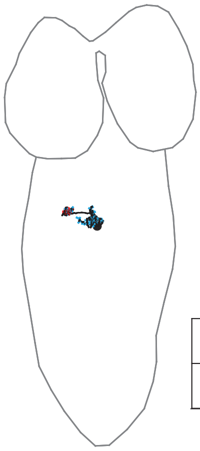

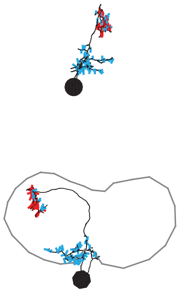

Inputs: #syn., (frac. syn)

	vdaB	v'ada	ddaC
Right	0 (0)	0 (0)	2 (0.027)
Left	3 (0.0405)	7 (0.0946)	7 (0.0946)

A02m (1) - a1R

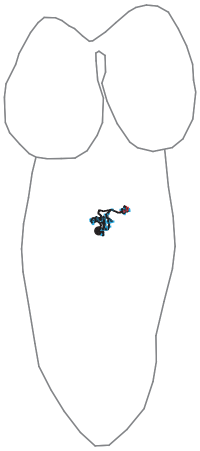

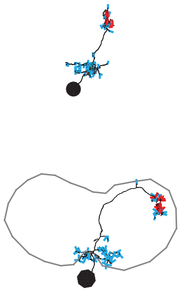

Inputs: #syn., (frac. syn)

	vdaB	v'ada	ddaC
Right	0 (0)	0 (0)	8 (0.103)
Left	1 (0.0128)	1 (0.0128)	3 (0.0385)

A09a - a1L (Basin-2)

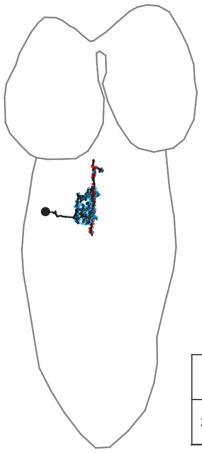

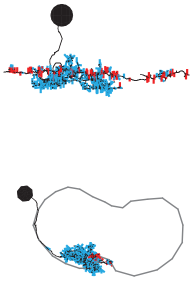

Inputs: #syn., (frac. syn)

	vdaB	v'ada	ddaC
Right	0 (0)	0 (0)	0 (0)
Left	2 (0.00678)	12 (0.0407)	4 (0.0136)

A09a - a1R (Basin-2)

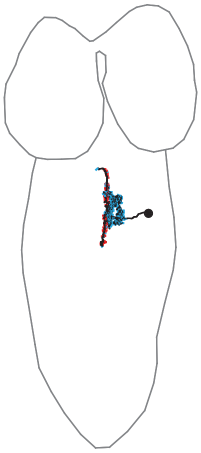

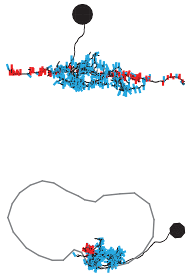

Inputs: #syn., (frac. syn)

	vdaB	v'ada	ddaC
Right	1 (0.00293)	11 (0.0323)	7 (0.0205)
Left	0 (0)	0 (0)	0 (0)

A09c - a1L (Basin-4)

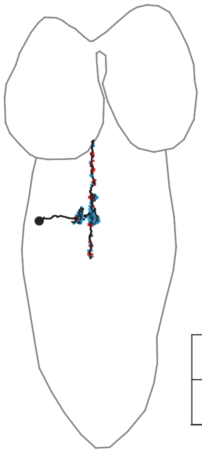

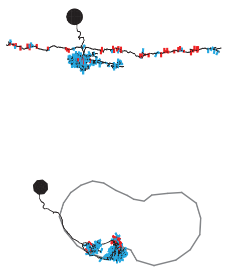

Inputs: #syn., (frac. syn)

	vdaB	v'ada	ddaC
Right	5 (0.0246)	0 (0)	0 (0)
Left	15 (0.0739)	10 (0.0493)	5 (0.0246)

A09c - a1R (Basin-4)

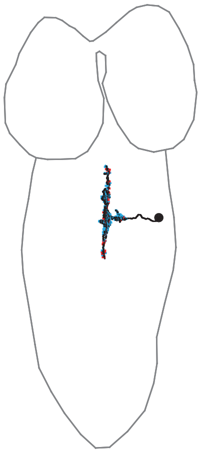

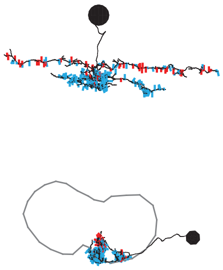

Inputs: #syn., (frac. syn)

	vdaB	v'ada	ddaC
Right	14 (0.0733)	19 (0.0995)	3 (0.0157)
Left	2 (0.0105)	0 (0)	0 (0)

A09l - a1L

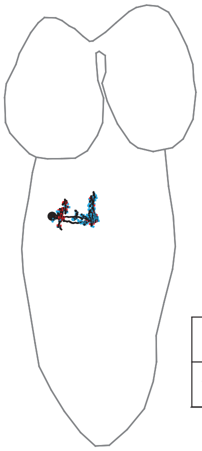

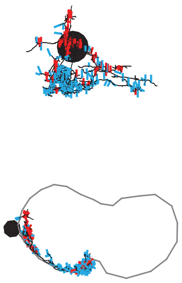

Inputs: #syn., (frac. syn)

	vdaB	v'ada	ddaC
Right	8 (0.0417)	0 (0)	1 (0.00521)
Left	18 (0.0938)	13 (0.0677)	6 (0.0312)

A09l - a1R

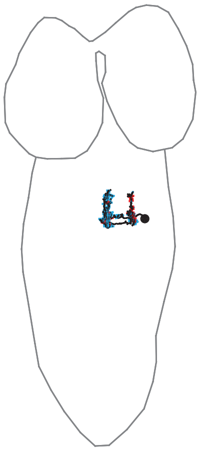

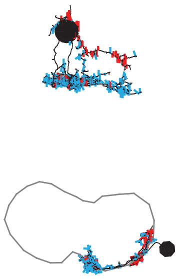

Inputs: #syn., (frac. syn)

	vdaB	v'ada	ddaC
Right	18 (0.0726)	29 (0.117)	16 (0.0645)
Left	4 (0.0161)	0 (0)	2 (0.00806)

A10a - a1L

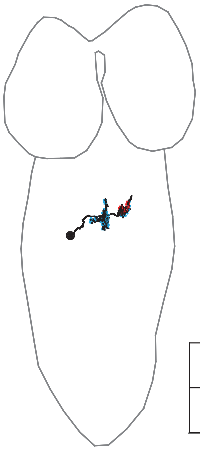

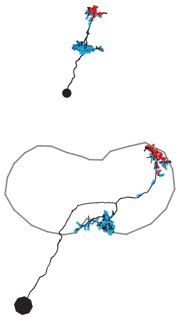

Inputs: #syn., (frac. syn)

	vdaB	v'ada	ddaC
Right	5 (0.0481)	5 (0.0481)	5 (0.0481)
Left	2 (0.0192)	1 (0.00962)	2 (0.0192)

A10a - a1R

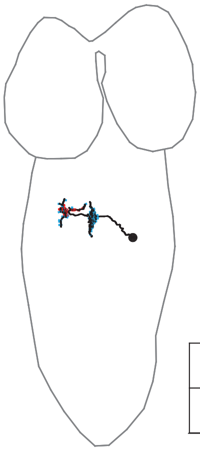

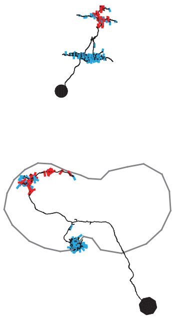

Inputs: #syn., (frac. syn)

	vdaB	v'ada	ddaC
Right	3 (0.0291)	0 (0)	0 (0)
Left	11 (0.107)	6 (0.0583)	5 (0.0485)

Regional Neurons

A02o - a2L

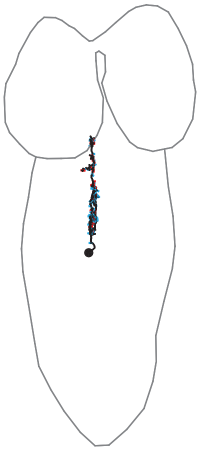

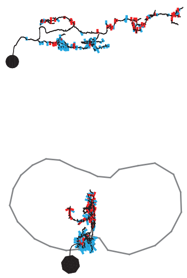

Inputs: #syn., (frac. syn)

	vdaB	v'ada	ddaC
Right	1 (0.0105)	0 (0)	1 (0.0105)
Left	1 (0.0105)	6 (0.0632)	7 (0.0737)

A02o - a2R

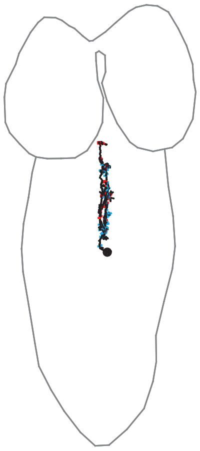

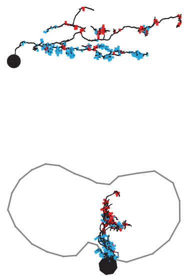

Inputs: #syn., (frac. syn)

	vdaB	v'ada	ddaC
Right	4 (0.029)	8 (0.058)	7 (0.0507)
Left	0 (0)	0 (0)	0 (0)

A09e - a3L

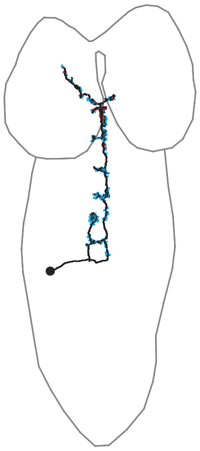

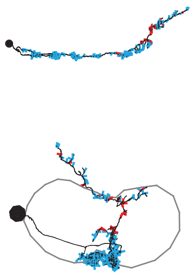

Inputs: #syn., (frac. syn)

	vdaB	v'ada	ddaC
Right	5 (0.0175)	3 (0.0105)	6 (0.021)
Left	12 (0.042)	10 (0.035)	11 (0.0385)

A09e - a3R

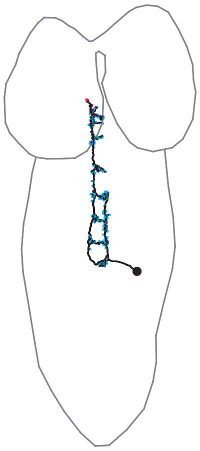

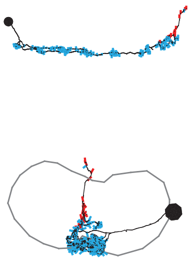

Inputs: #syn., (frac. syn)

	vdaB	v'ada	ddaC
Right	7 (0.0255)	6 (0.0218)	14 (0.0509)
Left	4 (0.0145)	1 (0.00364)	9 (0.0327)

A09o - a3L

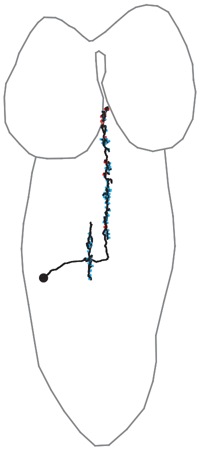

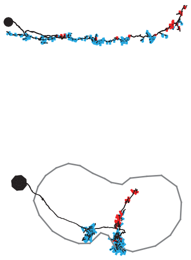

Inputs: #syn., (frac. syn)

	vdaB	v'ada	ddaC
Right	0 (0)	3 (0.0214)	3 (0.0214)
Left	0 (0)	0 (0)	0 (0)

A09o - a3R

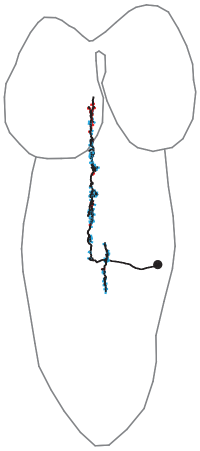

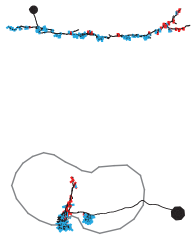

Inputs: #syn., (frac. syn)

	vdaB	v'ada	ddaC
Right	0 (0)	0 (0)	0 (0)
Left	2 (0.0152)	4 (0.0303)	3 (0.0227)

Descending Neurons

SeIN138 L

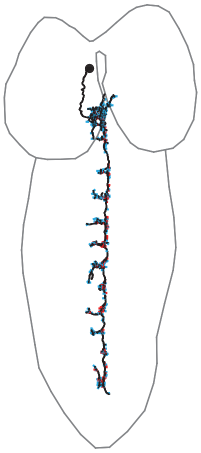
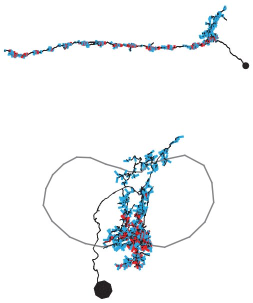

Inputs: #syn., (frac. syn)

	vdaB	v'ada	ddaC
Right	0 (0)	1 (0.00258)	2 (0.00515)
Left	4 (0.0103)	5 (0.0129)	3 (0.00773)

SeIN138 R

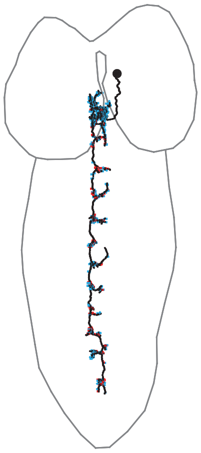
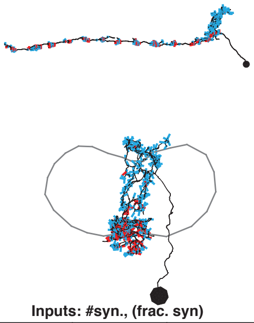

Inputs: #syn., (frac. syn)

	vdaB	v'ada	ddaC
Right	4 (0.00978)	7 (0.0171)	5 (0.0122)
Left	0 (0)	2 (0.00489)	2 (0.00489)

Ascending Neurons

A08n - a8L

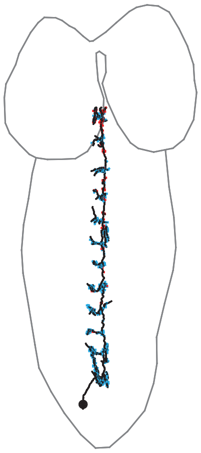
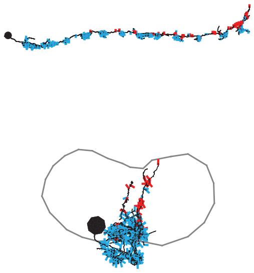

Inputs: #syn., (frac. syn)

	vdaB	v'ada	ddaC
Right	1 (0.002)	4 (0.00802)	13 (0.0261)
Left	2 (0.00401)	4 (0.00802)	7 (0.014)

A08n - a8R

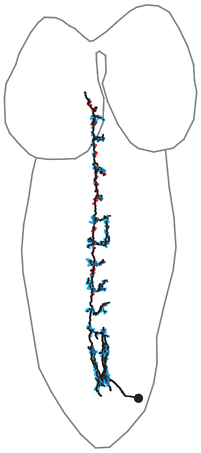
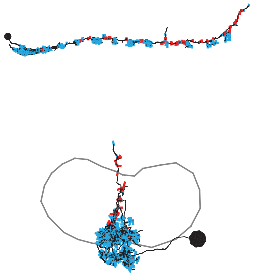

Inputs: #syn., (frac. syn)

	vdaB	v'ada	ddaC
Right	5 (0.011)	4 (0.00879)	10 (0.022)
Left	1 (0.0022)	2 (0.0044)	8 (0.0176)

A09q - a7L

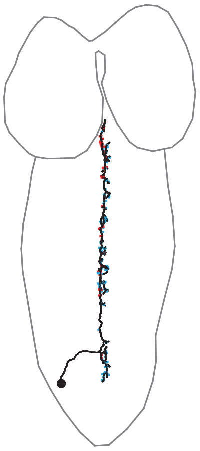
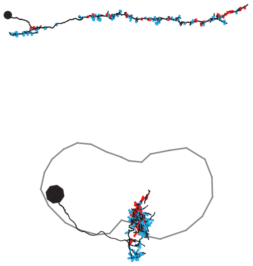

Inputs: #syn., (frac. syn)

	vdaB	v'ada	ddaC
Right	2 (0.0208)	1 (0.0104)	0 (0)
Left	0 (0)	0 (0)	0 (0)

A09q - a7R

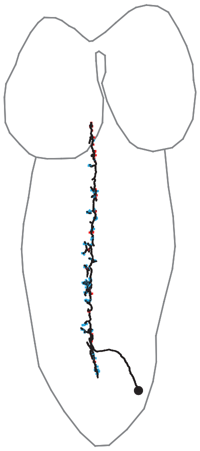
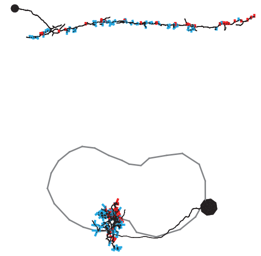

Inputs: #syn., (frac. syn)

	vdaB	v'ada	ddaC
Right	0 (0)	0 (0)	1 (0.0122)
Left	0 (0)	2 (0.0244)	2 (0.0244)

TePn05 L (incomplete)

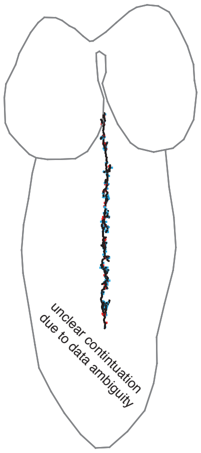

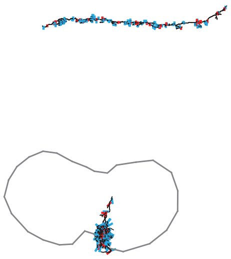

Inputs: #syn., (frac. syn)

	vdaB	v'ada	ddaC
Right	0 (0)	1 (0.00885)	2 (0.0177)
Left	1 (0.00885)	0 (0)	0 (0)

TePn05 R

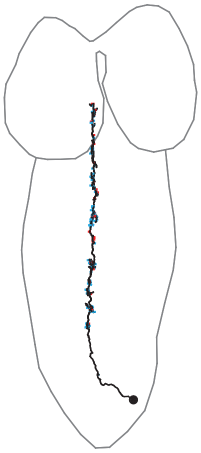

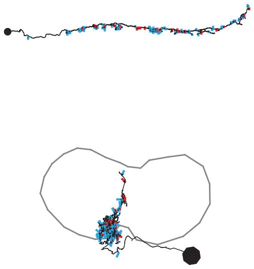

Inputs: #syn., (frac. syn)

	vdaB	v'ada	ddaC
Right	1 (0.00962)	0 (0)	0 (0)
Left	3 (0.0288)	2 (0.0192)	2 (0.0192)

TePn18 L

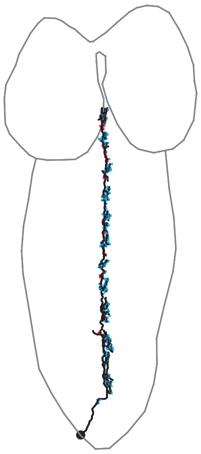

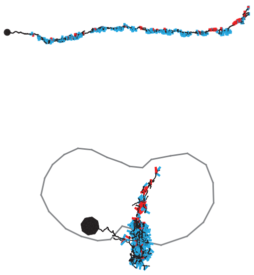

Inputs: #syn., (frac. syn)

	vdaB	v'ada	ddaC
Right	4 (0.0113)	11 (0.0312)	7 (0.0198)
Left	1 (0.00283)	0 (0)	0 (0)

TePn18 R

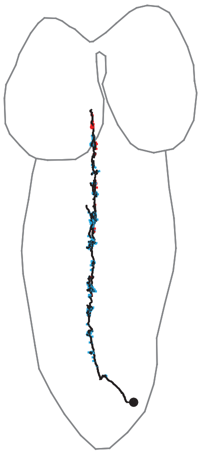

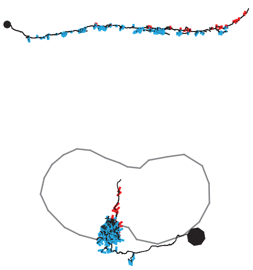

Inputs: #syn., (frac. syn)

	vdaB	v'ada	ddaC
Right	0 (0)	0 (0)	1 (0.00495)
Left	5 (0.0248)	12 (0.0594)	15 (0.0743)
